# Supplementary material for: Risk factors for sacrococcygeal pilonidal sinus: a systematic review and meta-analysis supplemented by genetic causal assessment
Source: Front Surg. 2026 Jan 7;12:1718589. doi: 10.3389/fsurg.2025.1718589 (PMC12819706; doi:10.3389/fsurg.2025.1718589)
Supplement: Supplementary file 2 [file Datasheet2.zip › Supplementary Data 2/MR_pipeline_p5e-6/funnel_plots/prot-c-3032_11_2.finngen_R12_L12_PILONIDALCYST.pdf]

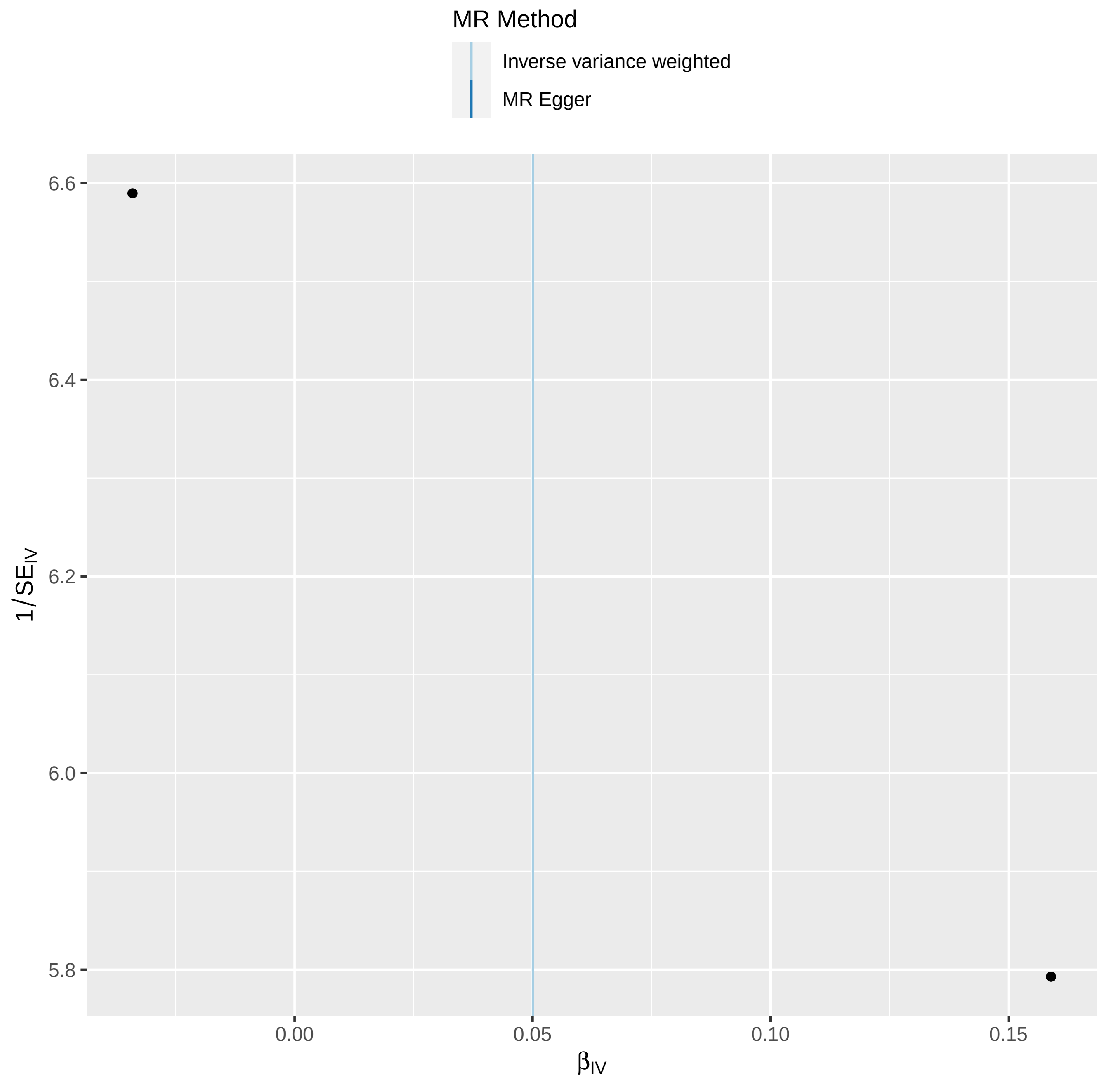
MR Method
Inverse variance weighted
MR Egger
6.6
6.4
6.2
6.0
5.8
1/SE_IV
0.00
0.05
0.10
0.15
β_IV
